# Supplementary material for: Pyruvate Dehydrogenase Kinase 1 inhibition mediated oxidative phosphorylation enhancement in cartilage promotes osteoarthritis progression
Source: BMC Musculoskelet Disord. 2023 Jul 20;24:597. doi: 10.1186/s12891-023-06585-6 (PMC10357736; doi:10.1186/s12891-023-06585-6)
Supplement: Supplementary file 1 — Additional file 1. [file 12891_2023_6585_MOESM1_ESM.zip › BP.Gsea.1653623667859/GOBP_AROMATIC_AMINO_ACID_FAMILY_METABOLIC_PROCESS.html]

Details for gene set GOBP\_AROMATIC\_AMINO\_ACID\_FAMILY\_METABOLIC\_PROCESS[GSEA]

|  || Dataset | 55457\_collapsed\_to\_symbols.cls.cls #OA\_versus\_normal.cls.cls #OA\_versus\_normal\_repos |
| Phenotype | cls.cls#OA\_versus\_normal\_repos |
| Upregulated in class | OA |
| GeneSet | GOBP\_AROMATIC\_AMINO\_ACID\_FAMILY\_METABOLIC\_PROCESS |
| Enrichment Score (ES) | 0.46793333 |
| Normalized Enrichment Score (NES) | 1.5429683 |
| Nominal p-value | 0.033254158 |
| FDR q-value | 0.31597337 |
| FWER p-Value | 1.0 |
Table: GSEA Results Summary

  

Fig 1: Enrichment plot: GOBP\_AROMATIC\_AMINO\_ACID\_FAMILY\_METABOLIC\_PROCESS      
 Profile of the Running ES Score & Positions of GeneSet Members on the Rank Ordered List

  

| SYMBOL | TITLE | RANK IN GENE LIST | RANK METRIC SCORE | RUNNING ES | CORE ENRICHMENT || 1 | QDPR | quinoid dihydropteridine reductase [Source:HGNC Symbol;Acc:HGNC:9752] | 112 | 0.897 | 0.1185 | Yes |
| 2 | KMO | kynurenine 3-monooxygenase [Source:HGNC Symbol;Acc:HGNC:6381] | 694 | 0.595 | 0.1578 | Yes |
| 3 | GSTZ1 | glutathione S-transferase zeta 1 [Source:HGNC Symbol;Acc:HGNC:4643] | 974 | 0.523 | 0.2104 | Yes |
| 4 | PARK7 | Parkinsonism associated deglycase [Source:HGNC Symbol;Acc:HGNC:16369] | 1406 | 0.442 | 0.2397 | Yes |
| 5 | TYR | tyrosinase [Source:HGNC Symbol;Acc:HGNC:12442] | 1547 | 0.421 | 0.2885 | Yes |
| 6 | HPD | 4-hydroxyphenylpyruvate dioxygenase [Source:HGNC Symbol;Acc:HGNC:5147] | 1681 | 0.402 | 0.3352 | Yes |
| 7 | TDO2 | "tryptophan 2,3-dioxygenase [Source:HGNC Symbol;Acc:HGNC:11708]" | 1783 | 0.387 | 0.3822 | Yes |
| 8 | TAT | tyrosine aminotransferase [Source:HGNC Symbol;Acc:HGNC:11573] | 1979 | 0.363 | 0.4186 | Yes |
| 9 | FAH | fumarylacetoacetate hydrolase [Source:HGNC Symbol;Acc:HGNC:3579] | 2264 | 0.330 | 0.4434 | Yes |
| 10 | KYNU | kynureninase [Source:HGNC Symbol;Acc:HGNC:6469] | 2502 | 0.303 | 0.4679 | Yes |
| 11 | HAAO | "3-hydroxyanthranilate 3,4-dioxygenase [Source:HGNC Symbol;Acc:HGNC:4796]" | 3209 | 0.229 | 0.4457 | No |
| 12 | PCBD1 | pterin-4 alpha-carbinolamine dehydratase 1 [Source:HGNC Symbol;Acc:HGNC:8646] | 3405 | 0.211 | 0.4604 | No |
| 13 | ATP7A | ATPase copper transporting alpha [Source:HGNC Symbol;Acc:HGNC:869] | 4814 | 0.086 | 0.3633 | No |
| 14 | PAH | phenylalanine hydroxylase [Source:HGNC Symbol;Acc:HGNC:8582] | 5012 | 0.072 | 0.3582 | No |
| 15 | TH | tyrosine hydroxylase [Source:HGNC Symbol;Acc:HGNC:11782] | 5374 | 0.042 | 0.3362 | No |
| 16 | IDO1 | "indoleamine 2,3-dioxygenase 1 [Source:HGNC Symbol;Acc:HGNC:6059]" | 7782 | -0.140 | 0.1692 | No |
| 17 | GCDH | glutaryl-CoA dehydrogenase [Source:HGNC Symbol;Acc:HGNC:4189] | 8180 | -0.170 | 0.1624 | No |
| 18 | THAP4 | THAP domain containing 4 [Source:HGNC Symbol;Acc:HGNC:23187] | 9330 | -0.258 | 0.1098 | No |
| 19 | HGD | "homogentisate 1,2-dioxygenase [Source:HGNC Symbol;Acc:HGNC:4892]" | 9669 | -0.289 | 0.1245 | No |
| 20 | DCT | dopachrome tautomerase [Source:HGNC Symbol;Acc:HGNC:2709] | 10995 | -0.429 | 0.0825 | No |
| 21 | TPH1 | tryptophan hydroxylase 1 [Source:HGNC Symbol;Acc:HGNC:12008] | 11223 | -0.460 | 0.1302 | No |
Table: GSEA details [plain text format]

  

Fig 2: GOBP\_AROMATIC\_AMINO\_ACID\_FAMILY\_METABOLIC\_PROCESS      
 Blue-Pink O' Gram in the Space of the Analyzed GeneSet

  

Fig 3: GOBP\_AROMATIC\_AMINO\_ACID\_FAMILY\_METABOLIC\_PROCESS: Random ES distribution      
 Gene set null distribution of ES for **GOBP\_AROMATIC\_AMINO\_ACID\_FAMILY\_METABOLIC\_PROCESS**
